# Beneficial early, harmful later: age-dependent effects of testicular factors on male mortality

**DOI:** 10.64898/2026.10.19.760267

**Published:** 2026-10-24

**Authors:** Laurence T. Maeyens, Nisi Jiang, Ziying Xu, Vivian Diaz, Randy Strong, Shangang Zhao, James F. Nelson

## Abstract

Men live shorter lives than women across nearly all human populations, a disadvantage that extends across most biological causes of death and that cannot be fully explained by behavioral or environmental differences. This suggests that fundamental biological mechanisms contribute to elevated male mortality. Our previous work implicated testicular factors as a major contributor, showing that prepubertal castration extends male lifespan to that of females in UM-HET3 mice, a genetically heterogeneous stock. However, it remains unknown when testicular factors begin to exert their detrimental effects on mortality. Here, we castrated UM-HET3 males before puberty (20 days), after puberty (60 days), or immediately before the typical rise in male mortality (240 days). Castration at all three ages prevented the early male mortality and increased median lifespan by 19.3%, 25.8%, and 32.9%, respectively. The comparable survival benefits of prepubertal and postpubertal castration indicate that exposure to the pubertal surge in testicular factors, including male sex hormones, is not responsible for establishing the male longevity disadvantage. Remarkably, castration at 240 days not only preserved the survival benefit of earlier castration but produced significantly greater survival than earlier castration, suggesting that testicular exposure before 240 days is actually beneficial. The immediate prevention of excess male mortality following castration at 240 days also indicates that the responsible testicular factors act rapidly and that their continued presence is necessary to sustain elevated male mortality. Longitudinal body-composition measurements further revealed that castration shifted males toward a female-like phenotype, characterized by reduced lean mass and continued fat accumulation into later life. Our findings reveal opposing age-dependent effects of testicular factors on survival and narrow the potential therapeutic window to later adulthood.

